# Barriers to physical activity in Spanish adolescents: gender differences

**DOI:** 10.3389/fpsyg.2026.1755136

**Published:** 2026-02-19

**Authors:** María Martín-Rodríguez, María IsabelBarriopedro, Jaime Prieto, José Antonio Santacruz-Lozano, Ángel Luis Clemente-Remón

**Affiliations:** 1 Grupo de Investigación Psicosocial en el Deporte, Facultad de Ciencias de la Actividad Física y del Deporte (INEF) Universidad Politécnica de Madrid, Madrid, Spain; 2 Department of Financial Economics and Accounting, Rey Juan Carlos University, Madrid, Spain; 3 Department of Biomedical Sciences, Alcalá University, Alcalá de Henares, Spain

**Keywords:** adolescents, barriers, gender, physical activity, secondary students

## Abstract

Given the concerning levels of physical inactivity among adolescents, particularly girls, the aim of this study was to identify gender differences in perceived barriers to physical activity (PA). A representative sample of 3,159 secondary school students in Spain (1,580 girls and 1,579 boys; mean age = 13.7 ± 0.7 years) completed the Perceived Barriers Scale to PA. The structure of the scale was examined to ensure its suitability for this population. While the original version included four types of barriers, two of them (contextual and safety) were found to be closely related. These were therefore combined into a single “environmental barriers” category, resulting in a revised model that fit the data well. The analysis showed that girls perceived more temporal and intrapersonal barriers to PA than boys. Additionally, regardless of gender, students who did not participate in extracurricular physical activities reported higher levels of all three types of barriers. These findings suggest that promotion strategies should pay special attention to the challenges faced by girls and less active adolescents in order to foster greater equity in physical activity.

## 1 Introduction

The physical, mental and cognitive health benefits of an active lifestyle during adolescence are well documented (Biddle et al., 2019; Carson et al., 2016; Poitras et al., 2016; Rodríguez-Ayllon et al., 2019). This evidence underpins the World Health Organization guidelines on physical activity (PA) and sedentary behavior. Adolescents should carry out at least an average of 60 min per day of moderate-to-vigorous intensity, mostly aerobic physical activity across the week, in addition to strength training 3 days per week. This includes physical education, sports and exercise, active travel, play and games (Bull et al., 2020).

However, approximately 8 out of 10 adolescents do not meet the current PA guidelines, with a greater prevalence of physical inactivity in girls (Araujo et al., 2024; Guthold et al., 2020). Thus, national and global action aimed at reducing gender inequalities and increasing access and opportunities for PA among those less active adolescents is a priority (Araujo et al., 2024; Guthold et al., 2020; Van Sluijs et al., 2021; World Health Organization [WHO], 2021).

Many factors contribute to adolescents’ PA behaviors. Bronfenbrenner’s (1979) ecological framework is one theoretical approach commonly mentioned as the most suitable to analyze how the physical, psychological, environmental and social factors influence the PA behavior of adolescents. The socio-ecological model which considers multiple levels of influence, ranging from individual to community, is particularly effective to promote multicomponent interventions most likely to be successful in adolescents (Martins et al., 2021; Perry et al., 2012; Van Sluijs et al., 2021).

A recent systematic review identifies the multilevel interplay of social, environmental and individual factors that contribute to the decline in PA from childhood through adolescence. Modifiable factors such as self-efficacy, motivation, academic workload and screen time emerged as key drivers of this trend (Habyarimana et al., 2025).

An updated systematic review of qualitative studies organized the main barriers and facilitators of adolescents’ PA around the five higher-order themes to highlight important relationships within the socio-ecological model dimensions and components: Individual factors such as motivation, self-efficacy and motor skills; social and relational factors related to family or friends; PA nature factors such as fun, school-based PA and physical education; life-course factors such as time and competing activities; and sociocultural and environmental factors such as the availability of PA facilities or programs (Martins et al., 2021).

Regarding the barriers for adolescent girls, the umbrella review conducted by Duffey et al. (2021) highlights the most frequently identified as the lack of time and the lack of support from peers, family and teachers. From a gender perspective, Guthold et al. (2022) explain that the strongest barriers to adolescent girls’ participation in PA are the values and lack of support of family and friends, perceptions of safety and fewer opportunities to be active.

Following the socio-ecological model, the Spanish version of the Scale of Perceived Barriers to PA (Zaragoza et al., 2011) presents four levels of barriers: intrapersonal, contextual, safety and temporal. The subsequent validation by Sevil et al. (2017) grouped the contextual and safety barriers in one single factor. This questionnaire has been previously used in the Spanish population (Sevil et al., 2017; Luque and del Villar, 2019; Delfa-De-La-Morena et al., 2022; Martín Rodríguez et al., 2024), and reported greater temporal and intrapersonal disliking barriers in females. Other countries have also observed that adolescents girls report more barriers to PA than boys (Cowley et al., 2021; Lazarowicz et al., 2021; Moore et al., 2023; Rosselli et al., 2020).

Regarding the influence between PA status on perceived barriers to PA, the least active adolescents perceive more barriers than more active peers (Delfa-De-La-Morena et al., 2022; Moore et al., 2023; Rosselli et al., 2020; Sevil et al., 2017).

Van Sluijs et al. (2021) aim to identify populations at risk of physical inactivity and understand its modifiable causes to inform intervention efforts. In Spain, girls and adolescents in secondary school are identified as physical inactivity risk groups (Delfa-De-La-Morena et al., 2022; Gasol Foundation Europa, 2023). Guthold et al. (2022) indicate that more research is needed to identify the barriers to PA participation in girls, as well as their interaction, across diverse contexts and cultures.

However, after a thorough review of the literature, no studies have researched these gender and PA status differences in barriers to PA in a representative sample of the Spanish population in secondary education. We hypothesized that girls score higher than boys in some barriers, and less active adolescents perceive more barriers than more active peers. Therefore, the objective of this study was to identify gender and PA status differences in the perceived barriers to PA in Spanish adolescents in secondary school.

## 2 Methods

### 2.1 Participants and study design

The sample design aimed to offer an adequate level of representativeness of the population of girls and boys enrolled in compulsory secondary education (ESO in its Spanish acronym) in Spain. The sample procedure was by simple random selection of class groups with proportional allocation to subgroups according to gender and secondary year. The sample was comprised of 3,159 school children from 1st, 2nd, 3rd, and 4th years of compulsory secondary education (Age mean = 13.7 SD ± 0.7 years), 1,580 self-identified girls and 1,579 self-identified boys, with a margin of error of ± 1.63%, a confidence interval of 95%, and variance for the most unfavorable case of *p* and *q* = 50. To ensure representativeness, participants were drawn from a diverse range of Autonomous Communities across Spain, covering regions from the north (e.g., Basque Country, Galicia, Cantabria, Navarre), center (e.g., Madrid, Castile and León), and south (e.g., Andalusia, Extremadura, Canary Islands). The highest representation was from Madrid, followed by the Basque Country, and Andalusia, which broadly reflects the population size and school density of these regions. Participants were classified as non-active (*n* = 457, 319 girls and 138 boys) or active (*n* = 2,702, 1,441 boys and 1,261 girls) considering if they did or did not participate in any extracurricular physical activity or sport (including going for walks) apart from school physical education classes.

The technique used was a survey via an on-line questionnaire using the REDCap platform, carried out with the collaboration of the school physical education staff. To this end the collaboration of the teaching staff was sought via the digital platforms of the Consejo COLEF (General Council of Physical and Sports Education). An informative introductory letter was sent to those teachers and schools that volunteered to participate in the study. Participants, and their parents when appropriate, were asked to read and sign a consent form. All procedures complied with the Declaration of Helsinki and were approved by the Universidad Politécnica de Madrid ethics committee board (registration number 2022-0861).

### 2.2 Instruments

The Spanish version of the Perceived Barriers Scale (Chinn et al., 1999) by Zaragoza et al. (2011) was used to evaluate barriers. This questionnaire consisted of 17 items graded on a Likert scale from 0 to 6. Zaragoza et al. (2011) grouped the 17 items into four factors that emerged from their factorial analysis; factors with eigenvalues ≥ 1.0 were retained, and a factor loading cutoff of 0.45 was considered to be significant. Intrapersonal disliking with 8 items related to self-efficacy, and motivation (e.g., “I don’t enjoy PA,” “I’m not good at physical activity and sports”); temporal constraints with 4 items (e.g., “I have too much school work”); safety reasons with 2 items (e.g., “Physical activity outdoors is not safe”); and Contextual constraints with 3 items related to lack of equipment and social support for PA (e.g., “There is no good place to do PA”).

### 2.3 Statistical analysis

The internal structure of the Perceived Barriers Scale (Zaragoza et al., 2011) was examined using confirmatory factor analysis (CFA). As data did not follow a normal distribution (Mardia’s coefficient > 5) analyses were performed using the maximum likelihood and robust estimation methods (Bentler, 2005). The Satorra-Bentler chi-square (S-B χ^2^) was computed with the following goodness of fit indices (Bentler, 2006): the comparative fit index (CFI), the normed fit index (NFI), and the root mean square error of approximation (RMSEA) and its 90% confidence interval. Values of the CFI and NFI close to 0.95 and values of the RMSEA below 0.06 indicate a good fit (Hu and Bentler, 1999). Convergent validity was analyzed via the calculation of the average variance extracted (AVE), considering values of AVE ≥ 0.50. Furthermore, discriminant validity was analyzed comparing the AVE of the factors with the square of the correlation between them (Fornell and Larcker, 1981). Internal consistency of the factors was assessed by composite reliability (CR), adopting CR ≥ 0.70 as the cut-off value (Hair et al., 2014). The corrected item-factor correlations were also computed to evaluate item homogeneity. Values greater than 0.40 were considered satisfactory.

To analyze the invariance of the Perceived Barriers Scale structure according to gender and PA status, a multi-group analysis was conducted following a series of hierarchically ordered steps. According to Byrne (2010) and Cheung and Rensvold (2002), for invariance to exist it is necessary to verify two criteria: (a) the measurement model should be adjusted to each group; and (b) to perform a multigroup analysis, it is necessary to examine the following invariance types: configural invariance (unconstrained model), metric invariance (weak invariance), and scalar invariance (strong invariance). The comparison between models was conducted using the Satorra-Bentler scaled difference test (Crawford and Henry, 2003) or the change in CFI. If the difference between the CFI values of two nested models is higher than 0.01 in favor of the less restrictive model, the more restrictive model should be rejected (Cheung and Rensvold, 2002).

The analysis of physical activity levels as a function of PA status for boys and girls was performed using a two-way between-subjects MANOVA. Effect size was estimated using partial η^2^, with values of approximately 0.01, 0.06, and 0.14 interpreted as small, medium, and large effects, respectively (Cohen, 1988). Analyses were conducted using the IBM SPSS 26.0 and EQS 6.6 software packages.

## 3 Results

The four-factor structure (M4F) proposed by Zaragoza et al. (2011) showed an acceptable fit (Table 1). However, the AVE values for the Contextual Barriers factor (0.453) and the Safety Barriers factor (0.431) were below the recommended threshold and lower than the squared correlation between these two factors (*r*^2^ = 0.681), indicating problems with both convergent and discriminant validity. The three-factor model (M3F), obtained by merging the items of these two dimensions into a single *Environmental Barriers factor*, yielded an acceptable fit (Table 1). Although the statistical fit indices decreased slightly compared to the original four-factor model, the three-factor model (M3F) was retained due to its parsimony and theoretical coherence [ΔS-Bχ^2^(3) = 65.5, *p* < 0.05]. All standardized factor loadings were statistically significant and above 0.50, with the exception of Item 17 (0.48), which was retained due to its theoretical relevance to the construct (Table 2). The AVE for the *Environmental Barriers factor* was 0.60, indicating that more than half of the variance in the items was explained by the factor. The AVE for the *Temporal Barriers factor* was 0.62 and for the *Intrapersonal Barriers factor* 0.53. Composite reliability (CR) values were 0.77, 0.87, and 0.90, respectively. All corrected item—total correlations exceeded 0.40 (Table 3).

**TABLE 1:** Model fit statistics for the measurement models and for the multi-group CFA across gender and PA status.

		S-B χ^2^	ML χ^2^	df	NFI	CFI	RMSEA	CI 90% RMSEA
M4F		966.17	1,614.29	113	0.933	0.940	0.049	0.046–0.052
M3F		1,030.2	1,719.04	116	0.928	0.936	0.050	0.047–0.053
**Gender M3F**								
	Girls	701.67	1,117.61	116	0.914	0.927	0.057	0.053–0.061
	Boys	465	817.88	116	0.927	0.944	0.044	0.040–0.048
**PA status M3F**								
	Yes	876.43	1,534.9	116	0.92	0.930	0.049	0.046–0.052
	No	268.85	347.68	116	0.908	0.945	0.054	0.045–0.062
**Invariance across gender M3F**								
	Configural	1,154.95	1,935.49	232	0.920	0.934	0.050	0.047–0.053
	Metric	1,174.01	1,969.56	246	0.918	0.934	0.049	0.046–0.052
	Scalar	1,268.59	2,075.56	260	0.918	0.935	0.050	0.047–0.052
**Invariance across PA status M3F**								
	Configural	1,236.7	1,882.58	232	0.922	0.936	0.052	0.050–0.055
	Metric	1,305.53	1,960.8	246	0.918	0.932	0.052	0.049–0.055
	Scalar	1,624.02	2,327.06	260	0.922	0.934	0.054	0.052–0.057

S-B χ^2^, Satorra–Bentler chi-square; df, degrees of freedom; ML χ^2^, maximum likelihood chi-square; CFI, Comparative Fit Index; NFI, Normed Fit Index; RMSEA, Root Mean Square Error of Approximation; CI, Confidence Interval.

**TABLE 2:** Standardized factor loadings and factor correlations for the three-factor model (M3F).

Item	Factors		
	Environmental	Temporal	Intrapersonal
I1	0.78	–	–
I2	0.78	–	–
I3	0.81	–	–
I4	0.72	–	–
I5	0.79	–	–
I6	0.75	–	–
I7	–	0.48	–
I8	–	0.67	–
I9	–	0.54	–
I10	–	–	0.57
I11	–	–	0.66
I12	–	–	0.53
I13	–	–	0.74
I14	–	–	0.89
I15	–	–	0.67
I16	–	–	0.56
I17	–	–	0.80

**TABLE 3:** Corrected item-total correlations (CITC), means and standard deviations.

Item	CITC	M	SD
I1	0.55	1.0	1.6
I2	0.54	1.0	1.7
I3	0.50	0.9	1.7
I4	0.58	1.1	1.8
I5	0.52	0.8	1.5
I6	0.62	2.0	2.0
I7	0.79	2.6	2.0
I8	0.69	1.8	1.8
I9	0.76	2.8	2.0
I10	0.75	0.8	1.7
I11	0.71	1.0	1.6
I12	0.77	0.8	1.7
I13	0.65	1.1	1.6
I14	0.45	1.1	1.7
I15	0.71	1.2	1.8
I16	0.78	0.7	1.5
I17	0.58	0.8	1.5

The fit of the three-factor model (Table 1) was acceptable for both girls (*N* = 1,580) and boys (*N* = 1,579), as well as for students who reported engaging (*N* = 2,702) or not engaging (*N* = 457) in extracurricular physical activity. The indices obtained supported the equivalence of the factor loading pattern (configural invariance) across gender and PA status. The constraint of equal factor loadings (metric invariance) and intercepts (scalar invariance) produced acceptable fit values, with changes in CFI < 0.01 (Table 1). Thus, both metric and scalar invariance across gender and PA status were supported.

Table 4 presents the descriptive analyses of perceived barriers as a function of extracurricular physical-sports activity status for girls and boys. There was a multivariate main effect of gender [*F*(3, 3,153) = 12.17, *p* < 0.001, η^2^ = 0.011] and of PA status [*F*(3, 3,153) = 66.63, *p* < 0.001, η^2^ = 0.060], but no significant interaction between gender and PA status on perceived barriers [*F*(3, 3,153) = 2.33, *p* = 0.072]. Univariate analyses showed that girls reported higher *Temporal Barriers* [*F*(1, 3,155) = 30.74, *p* < 0.001, η^2^ = 0.010] and higher *Intrapersonal Barriers* [*F*(1, 3,155) = 7.14, *p* = 0.008, η^2^ = 0.002] than boys, whereas no significant gender differences were found for *Environmental Barriers* [*F*(1, 3,155) = 0.77, *p* = 0.381]. In addition, students who did not engage in any extracurricular physical-sports activity PA (non-active PA status) perceived higher *Environmental Barriers* [*F*(1, 3,155) = 88.25, *p* < 0.001, η^2^ = 0.027], higher *Temporal Barriers* [*F*(1, 3,155) = 5.97, *p* = 0.015, η^2^ = 0.002], and higher *Intrapersonal Barriers* [*F*(1, 3,155) = 180.55, *p* < 0.001, η^2^ = 0.054] than those who did participate in any extracurricular physical-sports activity PA (active PA status).

**TABLE 4:** Perceived barriers as a function of gender and PA status.

Factors		Girls			Boys			PA status	
		Yes	No	Total	Yes	No	Total	Yes	No
Environmental	*M*	0.92	1.50	1.04	0.84	1.47	0.89	0.88	1.49
	SD	1.14	1.41	1.22	1.15	1.35	1.18	1.14	1.39
Temporal	*M*	2.42	2.80	2.50	2.10	2.14	2.10	2.25	2.60
	SD	1.61	1.77	1.65	1.60	1.76	1.61	1.61	1.79
Intrapersonal	*M*	0.86	1.80	1.05	0.75	1.56	0.82	0.80	1.73
	SD	1.14	1.48	1.27	1.14	1.44	1.19	1.14	1.47
	*N*	1,261	319	1,580	1,441	138	1,579	2,702	457

## 4 Discussion

The objective of this study was to identify gender and PA status differences in the perceived barriers to PA in a representative sample of the Spanish population in secondary school.

To this end the Perceived Barriers Scale by Zaragoza et al. (2011), was applied, having been previously used with an adolescent population in Spain, and was subjected to a confirmatory factor analysis (CFA) to study its internal structure, and average variance extracted (AVE) was utilized to examine its convergent validity.

Although the four-factor structure proposed by Zaragoza et al. (2011) revealed an acceptable fit, the contextual and safety barriers factors showed conceptual overlap and limited discriminant validity. Therefore, their combination into a single Environmental Barriers factor was recommended. The resulting three-factor model showed a satisfactory fit and was preferable due to its parsimony and improved conceptual clarity. This approach is consistent with the findings of Sevil et al. (2017), who found that the safety barriers factor could be grouped with contextual barriers based on their factorial behavior.

The analysis showed no effect of gender on this *Environmental Barriers Factor*. When comparing these results with previous studies using safety barriers factor and contextual barriers factor separately, we found some disparity in the results. This coincides with Delfa-De-La-Morena et al. (2022) who found no gender effect in safety or contextual barriers, while Martín Rodríguez et al. (2024) found that the girls presented higher scores in safety and contextual barriers. This variability suggests the suitability of using the *Environmental Barriers factor*, with a significantly better fit than the two separate factors of contextual and safety barriers originally proposed by Zaragoza et al. (2011).

This *Environmental Barriers factor* of the Perceived Barriers Scale (Zaragoza et al., 2011) has strengths and limitations that should be taken into account when interpreting the findings. This questionnaire includes items related to lack of good or safe places nearby, equipment, social support from peers and family to practice autonomous PA in outdoors spaces, assessing a part related to environmental barriers most frequently cited by adolescent girls (Duffey et al., 2021), taking into account that girls were more likely than boys to perceive a lack of convenient and safe places nearby as barriers to PA (Ricardos et al., 2022). On the other hand, it did not include specific items related to organized programs guided by teachers or trainers which may be considered as a limitation and also should be addressed in future studies. This could explain why previous studies which had considered this type of practice indicated greater environmental barriers for girls (Cowley et al., 2021; Jongenelis et al., 2018; Guthold et al., 2022; Ricardos et al., 2022).

One key conclusion that emerges is that adolescent girls presented greater temporal and intrapersonal barriers to PA. The results identify gender differences in the *Temporal Barriers* and *Intrapersonal Barriers* factors, both being greater in the girls which coincides with previous studies that used the Perceived Barriers Scale (Zaragoza et al., 2011) in different Spanish population groups (Delfa-De-La-Morena et al., 2022; Luque and del Villar, 2019; Martín Rodríguez et al., 2024; Sevil et al., 2017) and agrees with the existing literature (Rosselli et al., 2020; Guthold et al., 2022; Lazarowicz et al., 2021; Moore et al., 2023).

The analysis of the possible effects between gender and PA status on perceived barriers, showed no significative interaction which contrasts with previous studies in which non-active girls reported more barriers than non-active boys (Delfa-De-La-Morena et al., 2022; Sevil et al., 2017). This finding is not entirely comparable since neither the range of the participants’ ages nor the educational stages evaluated were the same.

Another relevant conclusion is that independently of gender those not engaged in in any extracurricular physical-sports activity (non-active PA status), reported greater temporal, environmental and intrapersonal barriers than those engaged in any extracurricular physical-sports activity PA (active PA status). These results coincide with those obtained in the Spanish population by Sevil et al. (2017) and those of Delfa-De-La-Morena et al. (2022) who also found a greater incidence of intrapersonal and contextual, though not temporal, barriers in the non-active group. Similarly in other countries, Moore et al. (2023) and Jongenelis et al. (2018) found that less active adolescents were more likely to encounter these barriers compared to more active peers. On the other hand, Fernández et al. (2017) found no correlation between PA status and perceived barriers. This variation in the results may be due to the different instruments used to assess the perceived barriers, the different ages or educational stages, as well as the different ways of assessing PA status, with most studies using the IPAQ questionnaire. In this study it was considered of interest to evaluate PA status as a function of whether or not subjects engaged in extracurricular physical education in Compulsory Secondary Education in Spain because increasing PA for those with lower PA levels provides more health benefits (Araujo et al., 2024).

Interestingly, the *Temporal Barriers factor* attained the highest scores in all the groups analyzed in this study. Lack of time has been shown as one of the most important barriers for adolescent girls (Duffey et al., 2021) and also a recurrent barrier for adolescents, with the increasing workload and academic demands being suggested as the main reason (Martins et al., 2021; Van Sluijs et al., 2021). This academic workload is a social key driver of PA decline in adolescence (Habyarimana et al., 2025). From a gender perspective, it is worth highlighting that the item related to homework does not specify if these tasks are academic or household chores or caring for others, traditionally attributed to the female gender which could be considered as a limitation and also should be addressed in future studies.

The *Intrapersonal Barriers factor* was, together with the temporal one, greater in girls and in the non-active group. This factor assesses aspects related to motivation and self-efficacy identified as individual key drivers in the decline of PA through adolescence (Habyarimana et al., 2025). Physical competence and motor skills, as well as tiredness and laziness are items included in this intrapersonal disliking barriers factor provided by the Perceived Barriers Scale (Zaragoza et al., 2011). However, the questionnaire used in our study did not include items related to body image, femininity or sociocultural gender roles and norms identified in previous studies as relevant barriers for girls (Araujo et al., 2024; Cowley et al., 2021; Duffey et al., 2021; Fernández et al., 2017; Moore et al., 2023; Rosselli et al., 2020) which may be considered as a limitation and should also be addressed in future studies.

From a gender perspective, several studies highlight that gender inequality was a multilevel factor, crossing all socioecological levels (Araujo et al., 2024; Cowley et al., 2021; Duffey et al., 2021; Guthold et al., 2022; Martín Rodríguez et al., 2024; Ricardos et al., 2022) considering that individual, environmental and social barriers are connected. It has been shown that gender, age, self-efficacy, and motivation are correlates associated with PA (Bauman et al., 2012). It is well known that some arguments that could explain the gender gap in PA are based on the intrinsic socio-cultural barriers that girls face to participate in PA, such as stereotypes or cultural acceptability, or simply the gender-specific lack of opportunities for access because PA is often a recreational choice with more options for, and tailored towards, boys which may disproportionately disadvantage girls. Consequently, the girls find more time-constraints and lack of support from peers. This societal influence on girls’ PA is intrinsic in society and beyond any traditional determinant of PA. As a result, girls may also have more disliking and less enjoyment from PA practice and less confidence in their sporting abilities (Araujo et al., 2024; Cowley et al., 2021; Duffey et al., 2021; Guthold et al., 2022; Martín Rodríguez et al., 2024; Ricardos et al., 2022).

Finally, another limitation should be stated. First of all, the use of subjective self-reported instruments may elicit errors related to respondent recall or desirability bias. Moreover, the use of different questionnaires in the literature results in different items and barrier groups being evaluated, thus impairing an accurate comparison of findings. However, the weight of the different items has not evaluated which should be considered in future studies. Indeed, our study did not include items related to screen time, currently one of the key factors for the PA decline in adolescence. Therefore, it would be interesting to design a more comprehensive questionnaire, connected with current barriers to PA in adolescence incorporating a gender perspective.

This study highlights the influence of gender on the diverse barriers to PA impacting Spanish adolescents from a socio-ecological perspective. In terms of applicability, these findings have practical implications. To reduce perceived barriers to physical activity among adolescents, particularly girls and non-active students, interventions should be implemented across multiple socio-ecological levels. It is essential to engage several stakeholders at different levels to incorporate a gender-responsive approach toward PA participation. At the school level, teachers and physical education programs should integrate more inclusive, enjoyable, and confidence-building activities, especially for girls, while ensuring equal access to facilities and opportunities. Increased PA opportunities in and out of schools, along with adjustments to academic schedules and demands could help address temporal barriers. At the community level, policymakers and local authorities should ensure the availability of safe, accessible, and appealing spaces for free-time physical activity. Furthermore, actions to enhance family and peer support for PA and sport, especially among girls, can help reduce intrapersonal and environmental barriers. Finally, developing gender-sensitive interventions tailored to adolescents’ needs and incorporating their voices could further promote equitable participation in PA during adolescence.

## Data Availability

The raw data supporting the conclusions of this article will be made available by the authors, without undue reservation.
